# Identifying nexilin as a central gene in neutrophil-driven abdominal aortic aneurysm pathogenesis

**DOI:** 10.1186/s10020-025-01157-x

**Published:** 2025-03-26

**Authors:** Bohan Yang, Yiyan Xu, Fengfei Yan, Cheng Peng, Ye Song, Song Han, Haiyang Wang

**Affiliations:** 1 Department of Vascular Surgery, The First Affiliated Hospital of Guangzhou Medical University, No. 151, Yanjiang West Road, Yuexiu District, Guangzhou, China; 2 Department of General Surgery, The First Affiliated Hospital of Harbin Medical University, Harbin, 150001 China

**Keywords:** Abdominal aortic aneurysm, Neutrophils, Weighted gene co-expression network analysis, Nexilin, Immune cell infiltration, Machine learning

## Abstract

**Objectives:**

Abdominal aortic aneurysm (AAA) is an inflammation-driven disease in which neutrophil infiltration is critical to its progression. This study aims to explore the molecular mechanisms behind neutrophil infiltration in AAA and identify key regulatory genes.

**Methods:**

We utilized weighted gene co-expression network analysis (WGCNA) and differential gene expression analysis to compare AAA and healthy abdominal aortic tissues. Functional enrichment analysis and a protein–protein interaction (PPI) network were constructed to understand gene functions. Machine learning algorithms were applied to identify key hub genes, followed by in vivo validation using an ApoE-/- mouse model.

**Results:**

Neutrophils, NK cells, and pDCs were significantly increased in AAA tissues. WGCNA identified 234 genes associated with neutrophil infiltration, of which 39 were significantly differentially expressed. Functional enrichment analysis highlighted roles in actin-related processes and pathways. Nexilin (NEXN) was consistently identified as a key hub gene negatively correlated with immune cell infiltration. In vivo validation confirmed that NEXN inhibits AAA progression in ApoE-/- mice by regulating immune cell infiltration.

**Conclusion:**

NEXN plays a crucial role in modulating neutrophil infiltration in AAA. These findings provide new molecular insights into AAA pathogenesis and suggest NEXN as a potential target for AAA therapy.

## Introduction

Abdominal aortic aneurysm (AAA) is a common and potentially life-threatening disease, causing over 150,000 deaths worldwide annually (Bulder et al. 2020). The clinical presentation of AAA is diverse, often asymptomatic in its early stages and frequently discovered incidentally during physical examinations or imaging studies (Martelli et al. 2023; Marine et al. 2021). However, as the disease progresses, the arterial wall may gradually expand and eventually rupture, leading to fatal hemorrhage with an extremely high mortality rate (De Santis et al. 2020; Silvestri et al. 2019). While substantial evidence indicates the role of environmental factors in the development of AAA, genetic factors have also been found to play a significant role in its progression. Understanding the pathological mechanisms of AAA is crucial for developing effective prevention and treatment strategies.

The pathogenesis of AAA is characterized by inflammation of the aortic wall leading to immune cell infiltration, including neutrophils, macrophages, B lymphocytes, and T lymphocytes (Anagnostakos and Lal 2021, Márquez-Sánchez and Koltsova, 2022, Cai et al. 2023). It increases the activity of proteases and cytokines, resulting in the degradation of extracellular matrix proteins, particularly collagen and elastin, subsequently weakening the aortic wall (Yuan et al. 2021, Hao et al. 2023, Lepelley et al. 2021). Additionally, apoptosis of vascular smooth muscle cells and weakening of the medial layer lead to vascular dilation and aneurysm formation (Zhao et al. 2022, Chen et al. 2023, Zhang et al. 2021). A significant issue in late-stage AAA is the presence of intraluminal thrombus, occurring in approximately 75% of patients, which contains various proteases, especially those derived from neutrophils (Wagenhäuser et al. 2023; Weiss et al. 2021). In summary, AAA is currently considered a chronic inflammatory disease, with neutrophils accumulating at the aneurysm site and within the intraluminal thrombus, releasing harmful substances that contribute to the destruction of the aortic wall.

Weighted Gene Co-expression Network Analysis (WGCNA) is a bioinformatics method used to construct gene co-expression networks and identify functionally related gene modules (Tang et al. 2023, Zhu et al. 2024). WGCNA builds gene co-expression networks by calculating the expression similarities between genes and employs modular analysis to identify gene sets associated with specific Biological Processes (BP) or cell types (De Sadeleer et al. 2022; Zheng et al. 2023). This method offers unique advantages in studying complex diseases, as it can reveal potential regulatory mechanisms and hub genes at a genome-wide level (Tao et al. 2022, Wang et al. 2022, Hou et al. 2022). In recent years, WGCNA has been widely applied in research on various diseases, including cancer, cardiovascular diseases, and neurodegenerative disorders (Yin et al. 2022, Lu et al. 2023). By systematically uncovering disease-related gene modules, WGCNA provides insights into their biological functions and potential mechanisms, offering a theoretical basis for molecular diagnosis and targeted therapy of diseases.

Machine learning, a vital branch of artificial intelligence, has shown immense potential in biomedical research in recent years. By training on and learning from large datasets, machine learning algorithms can automatically identify patterns and trends, thereby enhancing the accuracy and efficiency of data analysis (Ravaut et al. 2021; Wang et al. 2022). In the context of gene selection and biomarker identification, machine learning can integrate gene expression data and clinical features to identify disease-related hub genes and molecular pathways (Zhong et al. 2022, Zhong et al. 2023). In this study, we applied various machine learning algorithms, including support vector machine (SVM), random forest (RF), and extreme gradient boosting (XGBoost), to further identify key hub genes associated with neutrophil infiltration in AAA. This approach improves the screening results' reliability and provides robust support for subsequent experimental validation.

Current therapies for AAA include medical management, surgical interventions (such as open repair and endovascular repair), and emerging approaches like gene and stem cell therapy (Harris 1988). Pharmacological treatments primarily involve statins, angiotensin-converting enzyme inhibitors, β-blockers, calcium channel blockers, and antiplatelet agents (Harris 1988). Despite advances in AAA treatment, its asymptomatic nature and variable growth rate associated with rupture risk pose challenges. Currently, there is a lack of reliable, widely accessible, and cost-effective biomarkers for AAA monitoring. As key players in immune-mediated inflammation, immune cells are closely related to the progression of AAA through their infiltration in the vessel wall (Márquez-Sánchez and Koltsova, 2022, Zhang et al. 2024, Xiong et al. 2022). Therefore, identifying biomarkers related to immunity and inflammation could provide theoretical and clinical guidance for the prevention and treatment of AAA. However, the role of genes associated with neutrophil infiltration in AAA remains largely unknown. This study aims to comprehensively explore genes related to immune infiltration using bioinformatics strategies and both in vivo and in vitro validation, assessing their diagnostic potential in AAA. We hope our findings can assist in diagnosing and treating AAA patients from an immunological perspective.

## Materials and methods

### Acquisition of mRNA and lncRNA expression data from AAA tissues and healthy tissues and batch effect removal

Download the data with the accession numbers GSE47472 and GSE57691 from the Gene Expression Omnibus (GEO) comprehensive gene expression database (https://www.ncbi.nlm.nih.gov/geo/). The GSE47472 dataset includes mRNA and lncRNA expression data of 14 AAA tissues and 8 healthy donor abdominal aorta tissues, while the GSE57691 dataset comprises mRNA and lncRNA expression data of 49 AAA tissues and 10 donor abdominal aorta tissues. Since both GSE47472 and GSE57691 datasets are based on the GPL10558 platform, the datasets were first annotated based on the platform's annotation information. Subsequently, the data were processed using the "Combat" package in R software version 4.2.2 to remove batch effects. Finally, the batch-effect-corrected datasets were merged for subsequent analysis.

### Analysis method for immune cell infiltration scoring in aneurysmal tissue based on single-sample gene set enrichment analysis (ssGSEA)

Firstly, based on the merged mRNA and lncRNA expression profile data, the "GSVA" program package in R software version 4.2.2 was used to conduct ssGSEA. This analysis is mainly utilized to evaluate the infiltration scores of different immune cells in each sample's immune microenvironment. Through this method, the degree of infiltration of various immune cells, such as T cells, B cells, macrophages, etc., in each sample can be quantified, providing important information on the immune microenvironment characteristics of AAA tissues.

### Utilizing the WGCNA package to construct and analyze gene co-expression networks and their association with neutrophil content

In constructing a co-expression network using the WGCNA package in R software, the first step involves conducting a clustering analysis of samples to assess the presence of obvious outlier samples. Subsequently, the automatic network construction function is utilized, and the pickSoftThreshold function in R is used to calculate the soft-thresholding power β. This step aims to convert co-expression similarities into adjacency. Next, gene modules are dynamically detected and identified through hierarchical clustering and the dynamic tree-cut function. The fourth step involves calculating each gene's module membership and significance and correlating these modules with the cell content of neutrophils. Lastly, gene information from modules highly correlated with neutrophils is extracted for further functional and pathway analysis to reveal their roles in BP.

### Methodology for differential gene expression analysis using the 'limma'

In R software version 4.2.2, differential gene analysis is performed using the "Limma" package. Initially, this method defines significantly differentially expressed genes by setting specific criteria (fold change ≥ 1.5 and adjusted* p*-value < 0.05). In the process, gene expression data is first standardized; then, the Limma package utilizes linear modeling and empirical Bayes methods to estimate gene expression differences. Subsequently, multiple testing corrections are conducted, a*nd p*-values are adjusted using the Benjamini–Hochberg method to control the false discovery rate. Ultimately, differentially expressed genes meeting the specified threshold are identified, providing candidate targets for subsequent biological function and pathway analysis.

### Methodology for gene ontology (GO) and Kyoto Encyclopedia of Genes and Genomes (KEGG) pathway enrichment analysis using 'clusterprofiler'

In genetic big data analysis, GO functional enrichment analysis consists of three components: BP, Cellular Components (CC), and Molecular Functions (MF), while KEGG analysis aims to explore which signaling pathways are associated with gene sets. Utilizing the "ClusterProfiler" package in R software enables enrichment analysis based on GO. Perform KEGG enrichment analysis using the SangerBox database (http://sangerbox.com/). Firstly, download the "c2 KEGG gene set" GMT file from the MSigDB database (https://www.gsea-msigdb.org/gsea/msigdb/index.jsp). Then, KEGG-based gene set enrichment analysis (GSEA) was performed using the "clusterProfiler" package, with a significance threshold set at a *p*-value of less than 0.05.

### Construction and visualization of PPI networks of target mRNA-encoded proteins based on STRING and GeneMANIA databases

In this study, we initially conducted a PPI analysis of the proteins encoded by the target mRNA using the STRING database (https://string-db.org/). A PPI score threshold of 0.4 was set to ensure the reliability of interactions. The analysis results were then imported into Cytoscape software for further processing and visualization, including removing isolated nodes that did not interact with other proteins. Furthermore, we also constructed a more extensive PPI network through GeneMANIA (http://genemania.org), a user-friendly online database to delve deeper into the protein interaction network associated with the target genes. This network reveals direct interaction relationships and demonstrates potential functional correlations, providing valuable insights into the roles of the target genes in BP.

### Application and integration of LASSO logistic regression, SVM-REF, and RF algorithms in biomedical data analysis

In this study, we employed three different machine-learning algorithms to analyze and process biomedical data. Firstly, LASSO logistic regression was implemented using the ‘‘glmnet’’ package in R software. This method utilizes L1 regularization for feature selection, effectively screening out features that significantly impact the target variable. Secondly, SVM-Recursive Feature Elimination (SVM-REF) was implemented through the ‘‘e1071’’ package. This method optimizes feature selection and estimates generalization error through REF (svmRFE function) and external cross-validation. Feature ranking was performed on all training sets using the Lapply function and the top. Features function was used with ten-fold cross-validation to determine the optimal gene signature with the lowest error rate. Finally, the RF algorithm, executed through the "randomForest" package, aggregates results across multiple decision trees for classification, regression, and feature selection, enhancing model accuracy and stability. This combined approach provides a powerful toolkit for the complex analysis and interpretation of biomedical data.

### Standardized culturing methods for cardiovascular-related cell lines

In this study, we utilized various cell lines for experiments, including human aortic endothelial cells (HUVEC, Lonza, Cat. No.: CC-2517), human aortic smooth muscle cells (HAoSMC, Lonza, Cat. No.: CC-2583), mouse vascular smooth muscle cells (MOVAS, ATCC, Cat. No.: CRL-2797), human umbilical vein endothelial cells (HUVEC, ATCC, Cat. No.: PCS-100-010), and human coronary artery smooth muscle cells (HCASMC, Lonza, Cat. No.: CC-2585). All cells were cultured at 37 °C in a 5% CO_2_ humidified incubator. The culture medium used was DMEM (Gibco, USA) supplemented with 10% fetal bovine serum (Gibco, USA) and 1% penicillin–streptomycin (Gibco, USA) to support cell growth and proliferation. This standardized culturing method ensured the repeatability and comparability of experimental data.

### Methods for lentivirus-mediated overexpression and CRISPR-Cas9 knockout of the NEXN gene and validation of its expression

The lentiviral vector system (GeneCopoeia, Catalog Number: LVP015) was utilized to overexpress NEXN (pLVX-IRES-ZsGreen1-NC/NEXN Vector). Furthermore, for the knockout of NEXN, we employed the CRISPR-Cas9 gene editing system (Thermo Fisher Scientific, Catalog Number: A36499) with the following guide RNA sequences:

uni-tracrRNA: AAACAGCAUAGCAAGUUAAAAUAAGGCUAG UCCGUUAUCAACUUGAAAAAGUGGCACCGAGUCG GUGCU; gRNA_nexn_Exon2_antisense: AAACCGCTGTTCATCTCCACCTCT; gRNA_nexn_Exon2_sense: TAGGAGAGGTGGAGATGAACAGCG. In the overexpression experiments, after lentiviral transduction of cells for 24 h, the medium was replaced with fresh culture medium, and cells were further cultured for 48 h to promote gene expression. For NEXN knockout, upon transfection with the CRISPR-Cas9 system, cells successfully knocked out were selected through antibiotic resistance-free selection and cloning techniques. To validate the knockout and overexpression, a qPCR assay kit (Thermo Fisher Scientific, Catalog Number: A25742) and Western blot (WB) assay kit (Thermo Fisher Scientific, Catalog Number: PI32106) were utilized to detect the expression levels of NEXN. This assessment allowed the evaluation of the effects of lentivirus-mediated overexpression and CRISPR-Cas9-mediated knockout. Furthermore, PCR and sequencing techniques were employed to verify the CRISPR-Cas9-mediated gene knockout, ensuring the accuracy and efficacy of the gene editing.

### Experimental method for evaluating the effects of NEXN overexpression and knockout on cell proliferation using the CCK-8 assay

The CCK-8 assay method assessed the impact of NEXN overexpression or knockdown on cell proliferation. Initially, genetically manipulated cells were seeded in a 96-well plate at a density of 5 × 10^3^ cells per well. Subsequently, 10 μL of CCK-8 reagent (Dojindo, Catalog Number: CK04) was added to each well, and the plate was then incubated at 37 °C in a cell culture incubator for 2 h. Following the incubation period, the absorbance of each well was measured at a wavelength of 450 nm using a microplate reader. Five replicate wells were set up for each experimental group to ensure the reliability and accuracy of the experimental data. Statistical analysis was performed by calculating and comparing the cell proliferation rates of different treatment groups to elucidate the specific effects of NEXN overexpression or knockdown on cell proliferation activity.

### Assessment of the impact of NEXN gene overexpression and knockout on cell migration ability using transwell cell migration assay

The Transwell migration assay was used to assess the impact of NEXN overexpression or knockdown on cell migration ability. Initially, treated cells (5 × 10^4^ cells) were suspended in 200 μL serum-free medium and seeded in the upper chamber of a Transwell insert (Corning, Catalog Number: 3422). The lower chamber was filled with 600μL medium containing 10% fetal bovine serum to induce cell migration. The incubator was set at 37 °C, and cells were incubated for 24 h. After incubation, cells in the upper chamber were removed, and any cells that did not migrate through the filter membrane were wiped off with a cotton swab. Subsequently, cells that migrated to the lower chamber were stained with 0.1% crystal violet. Stained cells were counted under a microscope, with three replicate wells set up for each experimental group to ensure data reliability. The impact of NEXN expression status on cell migration ability could be quantitatively evaluated by comparing the number of migrated cells in different treatment groups.

### Evaluation of the impact of NEXN gene overexpression and knockout on cell invasion ability using matrigel invasion assay

The Matrigel invasion assay evaluated the impact of NEXN overexpression or knockdown on cell invasion capability. Initially, Matrigel (BD Biosciences, Catalog Number: 356234) was precoated in the upper chamber of a Transwell insert and incubated at 37 °C for 1 h to allow the Matrigel layer to solidify. Subsequently, treated cells (5 × 10^4^ cells) were suspended in 200 μL serum-free medium and seeded in the Matrigel-coated upper chamber. The lower chamber was filled with 600 μL medium containing 10% fetal bovine serum to act as a chemoattractant to promote cell invasion. Following seeding, cells were incubated at 37 °C for 24 h. After incubation, non-invading cells in the upper chamber were removed, and any cells that did not penetrate the Matrigel layer were gently wiped off with a cotton swab. Subsequently, cells that invaded the lower chamber were stained with 0.1% crystal violet, and stained cells were counted under a microscope. Three replicate wells were set up for each experimental group to ensure experimental accuracy and reproducibility. This experimental method allows for the quantitative analysis of the specific impact of NEXN expression status on cell invasion capability.

### Analysis of NEXN expression in immune cell subsets in aneurysmal tissue using flow cytometry

To quantitatively analyze the expression of NEXN in different immune cell subtypes (such as neutrophils, NK cells, etc.) in AAA tissues, this study employed fluorescence-activated cell sorting (FACS) technology. Initially, single-cell suspensions were extracted from abdominal aorta tissues of AAA and control groups using the Single Cell Isolation Kit from Miltenyi Biotec (Catalog Number: 130–098-608). Subsequently, cell labeling was performed using specific immune cell marker antibodies, including neutrophil marker antibody (BioLegend, Catalog Number: 320606) and NK cell marker antibody (BioLegend, Catalog Number: 339916), along with the antibody targeting NEXN (Abcam, Catalog Number: ab228805) for dual staining. Cell detection was conducted using the FACSCanto II flow cytometer from BD Biosciences. Data analysis was performed using flow cytometry analysis software such as FlowJo, displaying bar graphs or line graphs to show the expression differences of NEXN in various immune cell subtypes, revealing its potential role in AAA development.

### Assessing the impact of NEXN on the development of AAA using the ApoE^−/−^ mouse model

An AAA model was constructed using ApoE^−/−^ mice (Saiye Biotechnology, C001507). Initially, the Alzet osmotic mini pump (Model 2004) was removed and soaked in sterile 37 °C saline for 40 h for preparation. Once the osmotic pump assembly was completed, 200 μL of AngII solution (concentration of 1 μg/kg/min) was injected into each pump. Experimental mice were anesthetized using isoflurane inhalation anesthesia, and the pre-filled osmotic pumps were subcutaneously implanted. The mice were divided into four groups, each consisting of 10 mice: a control group (subcutaneous implantation of saline), an AAA model group (subcutaneous implantation of AngII solution), a NEXN overexpression group (subcutaneous implantation of AngII solution and slow virus vector overexpressing NEXN), and a NEXN knockout group (subcutaneous implantation of AngII solution and CRISPR-Cas9-mediated knockout of NEXN). Postoperative monitoring of the mice's condition was conducted continuously, and the mice were euthanized four weeks postoperatively to extract intact aortic tissue for subsequent experimental analysis. The animal experiments in this study were approved by the Animal Ethics Committee of the First Affiliated Hospital of Guangzhou Medical University) and conducted according to relevant ethical guidelines.

### Quantitative analysis of NEXN and other genes expression in AAA tissues using qRT-PCR technique

Tissue samples were first ground into powder, or cells were collected and lysed using RNA-easy lysis buffer (Qiagen, Catalog Number: 74106) for complete lysis to detect the expression of NEXN and other target genes in AAA tissues. Subsequently, RNase-free ddH_2_O was added for mixing, followed by centrifugation at 12,000 g for 15 min. The supernatant was collected, and an equal volume of isopropanol (Sigma-Aldrich, Catalog Number: I9516) was added, mixed, and centrifuged again at 12,000 *g* for 10 min. The supernatant was discarded, and 75% ethanol was added to the pellet. After gentle mixing, centrifugation was performed at 8000 *g* for 3 min, the supernatant was removed, and an appropriate amount of RNase-free ddH_2_O was added to dissolve the RNA pellet. The concentration and purity of RNA were measured using a UV1800 instrument (Shimadzu, Japan). An appropriate amount of RNA was mixed with reverse transcription primer and incubated at 65 °C on a PCR machine for 5 min, followed by rapid cooling. Buffer, dNTPs, RNA inhibitor, and reverse transcriptase were added, mixed, and then incubated at 42 °C on the PCR machine for 60 min, followed by a final incubation at 80 °C for 5 min to deactivate the reverse transcriptase. The qPCR reaction system consisted of: 2 × qPCR Mix 12.5 μL, 7.5 μM gene-specific primers 2.0 μL, reverse transcription product 2.5 μL, and ddH_2_O 8.0μL. The primers used were as follows: NEXN primers: Forward: 5′—AGGAGGAGGAGGAGGAGG—3′, Reverse: 5′—TCCTCCTCCTCCTCCTCC-3' Reference gene GAPDH primers: Forward: 5′—GTCTCCTCTGACTTCAACAGCG—3′, Reverse: 5′—ACCACCCTGTTGCTGTAGCCAA—3′ The qPCR cycling conditions were as follows:—Initial duration at 95 °C for 10 min—40 cycles consisting of denaturation at 95 °C for 15 s and annealing/extension at 60 °C for 60 s—Melting curve analysis ramping from 75 °C to 95 °C, increasing by 1 °C every 20 s. For qPCR analysis, specific NEXN and reference gene GAPDH primers were used. The 2^−ΔΔCT^ method was employed for normalizing NEXN expression using GAPDH as the reference gene for analysis.

### Methodology for detecting the expression of NEXN and related proteins in AAA tissues and cells using WB technique

Samples were initially lysed by adding a lysis buffer composed of RIPA buffer (Thermo Fisher Scientific, Catalog Number: 89900), protease inhibitor (Roche, Catalog Number: 11836153001), and phosphatase inhibitor (Roche, Catalog Number: 04906845001) to ensure complete sample lysis, to detect the expression levels of NEXN and related proteins in AAA tissues and cells. The lysates were centrifuged at 12,000 g for 10 min, and the supernatant was collected for further analysis. The BCA method determined the protein concentration (Pierce, Catalog Number: 23227). The proteins were mixed with sample buffer for SDS-PAGE (Bio-Rad, Catalog Number: 1610747) and boiled at 100 °C for 5 min. Subsequently, the samples were loaded onto an SDS-PAGE gel and electrophoresed at 80 V voltage for separation. After electrophoresis, the proteins were transferred to a PVDF membrane (Millipore, Catalog Number: IPVH00010) using a 200 mA current for 3 h. Following the transfer, the PVDF membrane was blocked with 5% skimmed milk (Bio-Rad, Catalog Number: 1706404) at room temperature for 1 h. Subsequently, primary antibodies against NEXN (Abcam, Catalog Number: ab228805, dilution 1:1000) and the housekeeping protein β-actin antibody (Cell Signaling Technology, Catalog Number: 4967, dilution 1:2000) were added and the membrane was incubated overnight at 4 °C. The following day, the membrane was washed thrice with PBST solution for 10 min each. Subsequently, a secondary antibody labeled with HRP (Cell Signaling Technology, Catalog Number: 7074, dilution 1:2000) was added, and the membrane was incubated at room temperature for 1 h. After incubation, the membrane was washed thrice with PBST for 10 min each. Finally, the membrane was visualized using an ECL detection reagent (Thermo Fisher Scientific, Catalog Number: 32106), and images were captured and analyzed using an Odyssey Fc Imager (LI-COR Biosciences, Model: Odyssey Fc).

### Statistical analysis

All experimental results are presented as mean ± standard deviation (Mean ± SD). For data conforming to a normal distribution, independent sample t-tests were used to compare two groups; the Mann–Whitney U test was employed for non-normally distributed data. Multiple group comparisons were conducted using one-way analysis of variance (ANOVA), followed by post hoc analysis using LSD-t tests. Pearson correlation analysis was utilized to assess the linear relationship between variables. Diagnostic efficacy evaluation was performed using receiver operating characteristic (ROC) curve analysis, calculating the area under the curve (AUC) to determine the sensitivity and specificity of NEXN in AAA diagnosis. All statistical analyses were carried out using SPSS 25.0 software (IBM), with the significance level set at *p* < 0.05 (denoted as *p < 0.05*,* **p < 0.01, ***p < 0.001).

### Ethics statement

This study involved human tissue samples and animal experiments, strictly adhering to ethical standards and international norms. All experiments involving human samples received approval from the institutional medical ethics committee, and all participants provided informed consent after fully understanding the research objectives and procedures. The collection and use of human samples strictly adhered to the ethical guidelines of the Helsinki Declaration. For the animal experiments, all procedures conducted using the ApoE^−/−^ mouse model were approved by the institution's animal ethics and usage committee. The experimental design fully considered the principles of the 3Rs (Replacement, Reduction, Refinement), ensuring the use of the minimum number of animals and alleviating animal suffering. All experimental procedures were carried out under strict aseptic conditions and executed by trained technicians to maximize animal welfare. Furthermore, all animals in the experiment received appropriate anesthesia and postoperative analgesia measures to minimize pain during the experimental process. After the experiments, all animals were humanely euthanized to minimize any discomfort and suffering. Additionally, this study strictly adhered to relevant laws and regulations to ensure the ethical and scientific integrity of the research.

## Results

### Identification of immune cell infiltration imbalance and neutrophil-related genes in AAA tissues

**Fig. 1:**
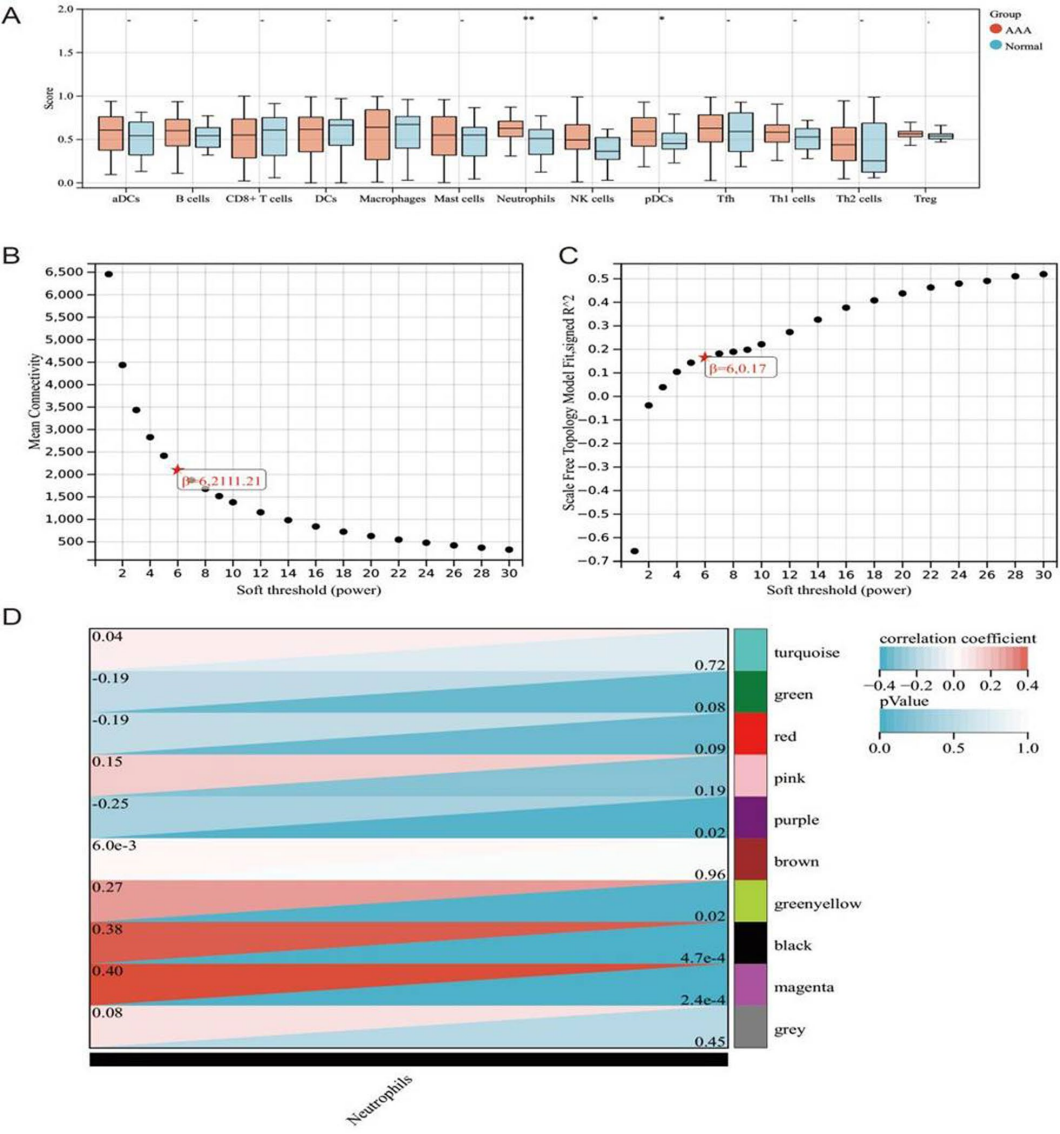
Screening of Genes Associated with Neutrophil Infiltration and WGCNA Analysis. **A** Comparison of immune cell infiltration scores between AAA and normal tissues (AAA group: n = 14, Normal group: n = 8). **B** Average connectivity at various soft threshold powers (power) in WGCNA analysis. **C** Topological overlap measure index (scale-free fit index) at various soft threshold powers (power) in WGCNA analysis. **D** Correlation analysis of gene co-expression modules associated with neutrophil infiltration

In this study, we conducted a detailed analysis of immune cell infiltration in AAA tissues and explored the molecular mechanisms associated with neutrophil infiltration. By comparing normal abdominal aortic tissues from donors, we found a significant increase in the infiltration scores of neutrophils, NK cells, and pDCs in AAA tissues, while no significant differences were observed in other immune cells (Fig. 1A). AAA is currently considered an inflammation-driven disease, as many related processes (such as macrophage, neutrophil, B-cell, T-cell infiltration, and activation of inflammatory pathways) have been found in humans and mice. The overactive inflammatory response damages the aortic media by releasing proteolytic enzymes, leading to vascular smooth muscle cell death and further promoting the development of AAA (Kan et al. 2021). In recent years, the importance of neutrophils in AAA has become apparent, as they are known to actively participate in processes such as oxidative stress, extracellular matrix degradation, adventitial inflammation, and intraluminal thrombus formation (Michel et al. 2010). Neutrophils not only passively accumulate in lesions during the development of AAA but also play an active role (Plana et al. 2020). Based on this background, we further explored the potential mechanisms related to neutrophil infiltration. Through WGCNA, we calculated the optimal soft threshold as 6 (Fig. 1B–C). Under this soft threshold condition, we divided genes from AAA and control tissues into 10 co-expression modules based on mRNA and lncRNA expression profiles. Correlation analysis indicated that the green-yellow, black, and magenta modules were positively correlated with neutrophil infiltration scores, while the purple module was negatively correlated with neutrophil infiltration scores (Fig. 1D). These modules collectively contained 234 genes, defined as genes related to neutrophil infiltration. In conclusion, our research findings demonstrate a significant imbalance in immune cell infiltration in AAA tissues, particularly a significant increase in neutrophils, and identified hub gene modules related to neutrophil infiltration through WGCNA, providing important clues for further research on the development mechanisms of AAA.

### Gene selection related to neutrophil infiltration and differential expression analysis in AAA tissues

**Fig. 2:**
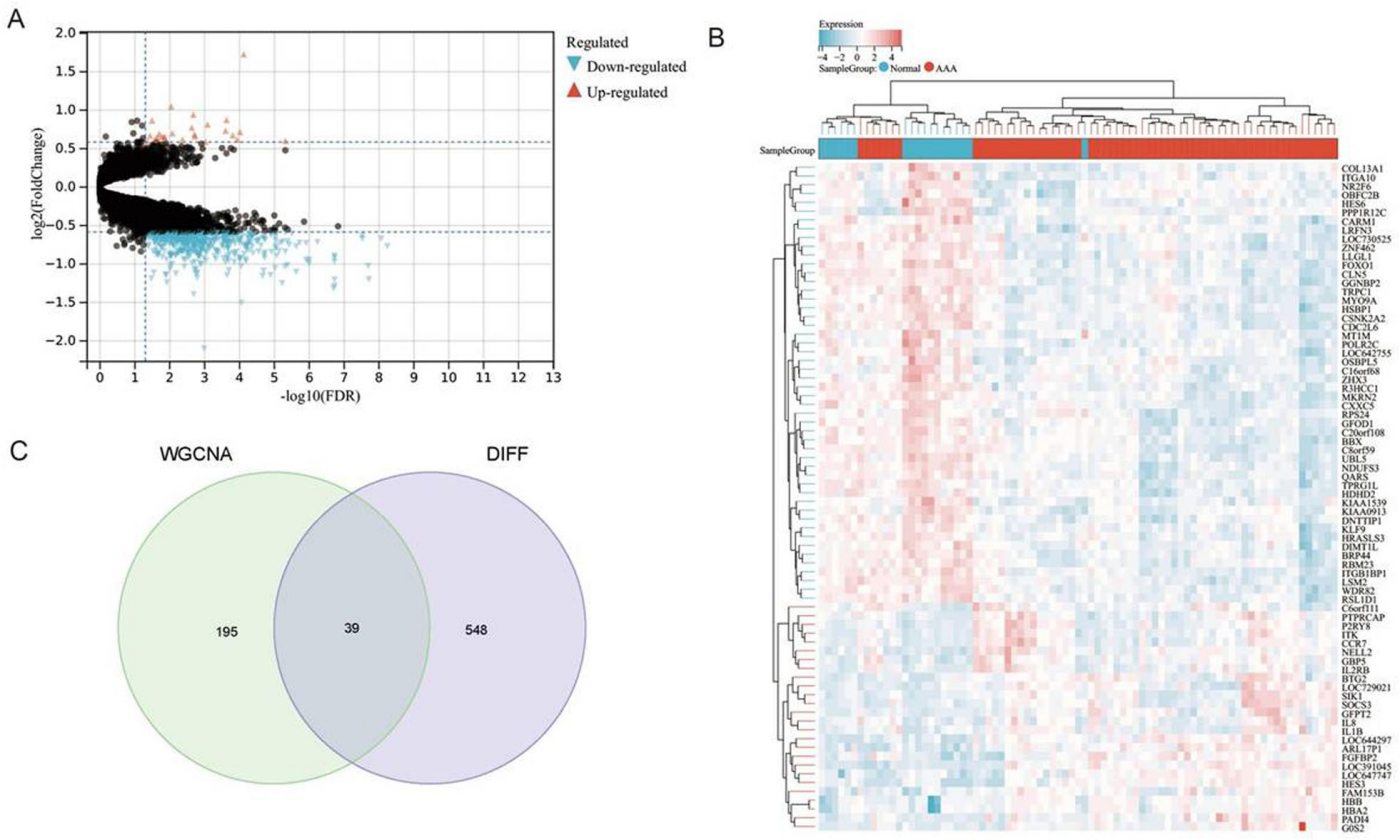
Screening of Differentially Expressed Genes Associated with Neutrophil Infiltration. Note: **A** Volcano plot of differentially expressed genes between AAA tissues and control tissues. **B** Heatmap of differentially expressed genes, showing the gene expression levels in AAA tissues compared to normal tissues (AAA group: n = 14, Normal group: n = 8). **C** Venn diagram of the intersection of genes selected from WGCNA and differential expression analyses

In the study of the pathogenesis of AAA, the infiltration of neutrophils is considered an important pathological process. Therefore, screening and analyzing genes related to neutrophil infiltration is of significant importance for understanding the pathophysiological mechanisms of AAA. Differential gene expression analysis between AAA and control tissues revealed that 26 genes were significantly upregulated in AAA tissues, while 561 genes were significantly downregulated (Fig. 2A–B). Subsequently, the intersection of these differentially expressed genes with 234 genes obtained from WGCNA led to the final selection of 39 differentially expressed genes related to neutrophil infiltration for further analysis (Fig. 2C). In summary, this study successfully identified differentially expressed genes associated with neutrophil infiltration, revealing significant changes in gene expression in AAA tissues and providing new molecular targets for a deeper understanding of the pathophysiological mechanisms of AAA.

### Functional enrichment analysis and PPI network reveal key molecular mechanisms related to neutrophil infiltration

**Fig. 3:**
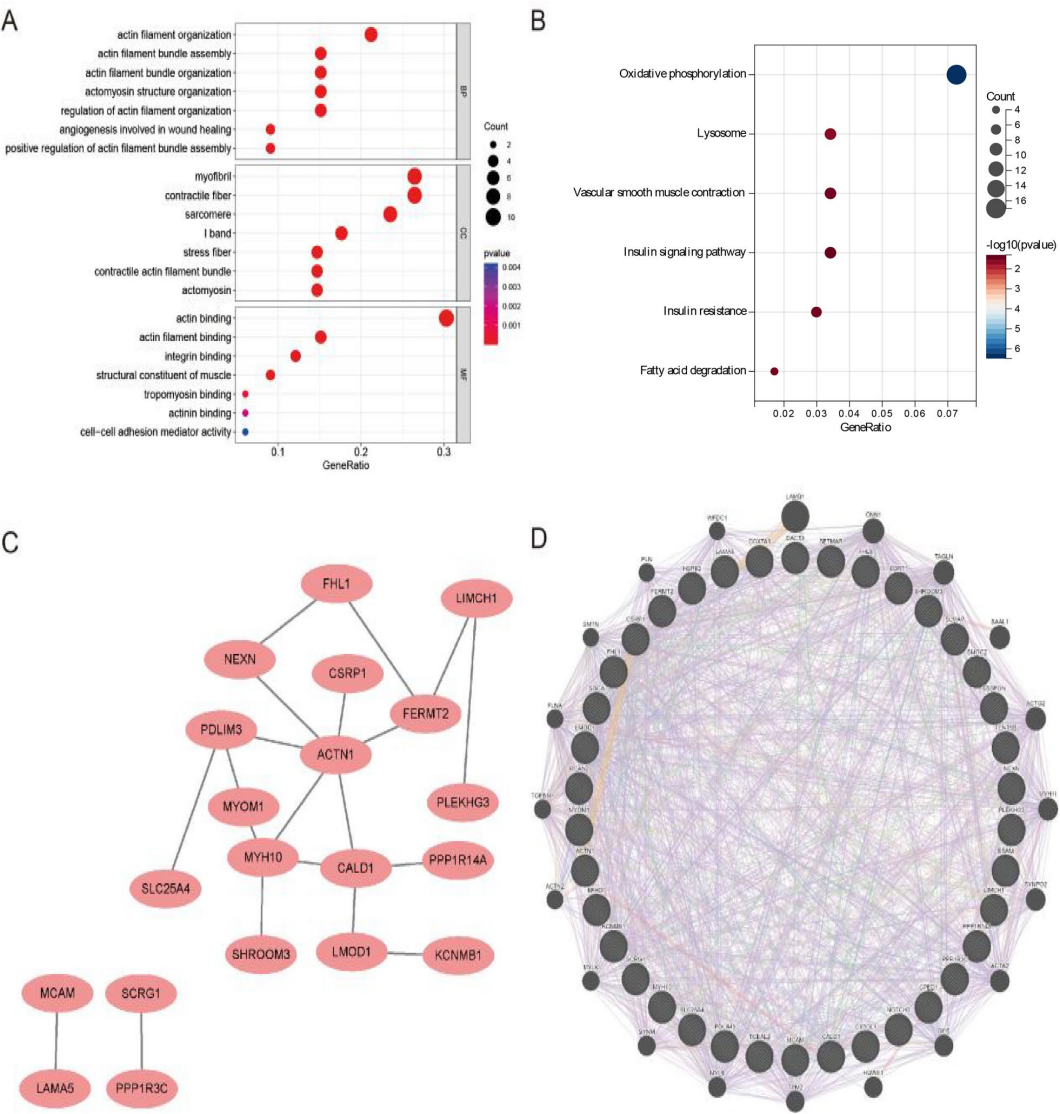
Functional Enrichment Analysis and PPI Network. **A** Results of GO enrichment analysis for BP, MF, and CC. **B** KEGG pathway enrichment analysis results. **C** PPI network of 39 genes generated according to the String database. **D** Protein interaction network related to the 39 genes displayed through GENEMANIA

To gain a deeper understanding of the functions of differentially expressed genes related to neutrophil infiltration and their roles in the pathogenesis of AAA, we conducted GO and KEGG enrichment analyses and constructed a PPI network. The GO-BP enrichment analysis indicated that these genes are mainly involved in processes such as actin filament organization, actin filament bundle assembly, actin filament bundle organization, actomyosin structure organization, regulation of actin filament organization, angiogenesis involved in wound healing, and positive regulation of actin filament bundle assembly (Fig. 3A). The GO-MF enrichment analysis revealed that these genes are associated with functions including actin binding, actin filament binding, integrin binding, structural constituent of muscle, tropomyosin binding, actinin binding, and cell–cell adhesion mediator activity (Fig. 3A). The GO-CC enrichment analysis indicated that the expression of these genes is associated with CC, such as myofibril, contractile fiber, sarcomere, I band, stress fiber, contractile actin filament bundle, and actomyosin (Fig. 3A). Given the potential for false positives in KEGG enrichment analysis, signaling pathways closely associated with AAA are presented, including oxidative phosphorylation, vascular smooth muscle contraction, lysosome, and fatty acid degradation pathways, which are highly enriched in AAA tissues (Fig. 3B). The PPI network constructed using the String database showed that after the removal of isolated nodes, the remaining network contained 20 proteins and 20 interactions (Fig. 3C). Among them, ACTN1, MYH10, and CALD1 are positioned at the core of the network due to their numerous interactions with other proteins. Further GENEMANIA analysis displayed 20 proteins interacting with the initial 39 proteins (Fig. 3D). In conclusion, functional enrichment analysis and PPI network construction revealed the significant roles of differentially expressed genes related to neutrophil infiltration in muscle-related BP and pathways within AAA tissues. These findings provide a new perspective for further research on the molecular mechanisms of AAA.

### Machine learning algorithms filter hub genes to reveal the molecular mechanisms of neutrophil

**Fig. 4:**
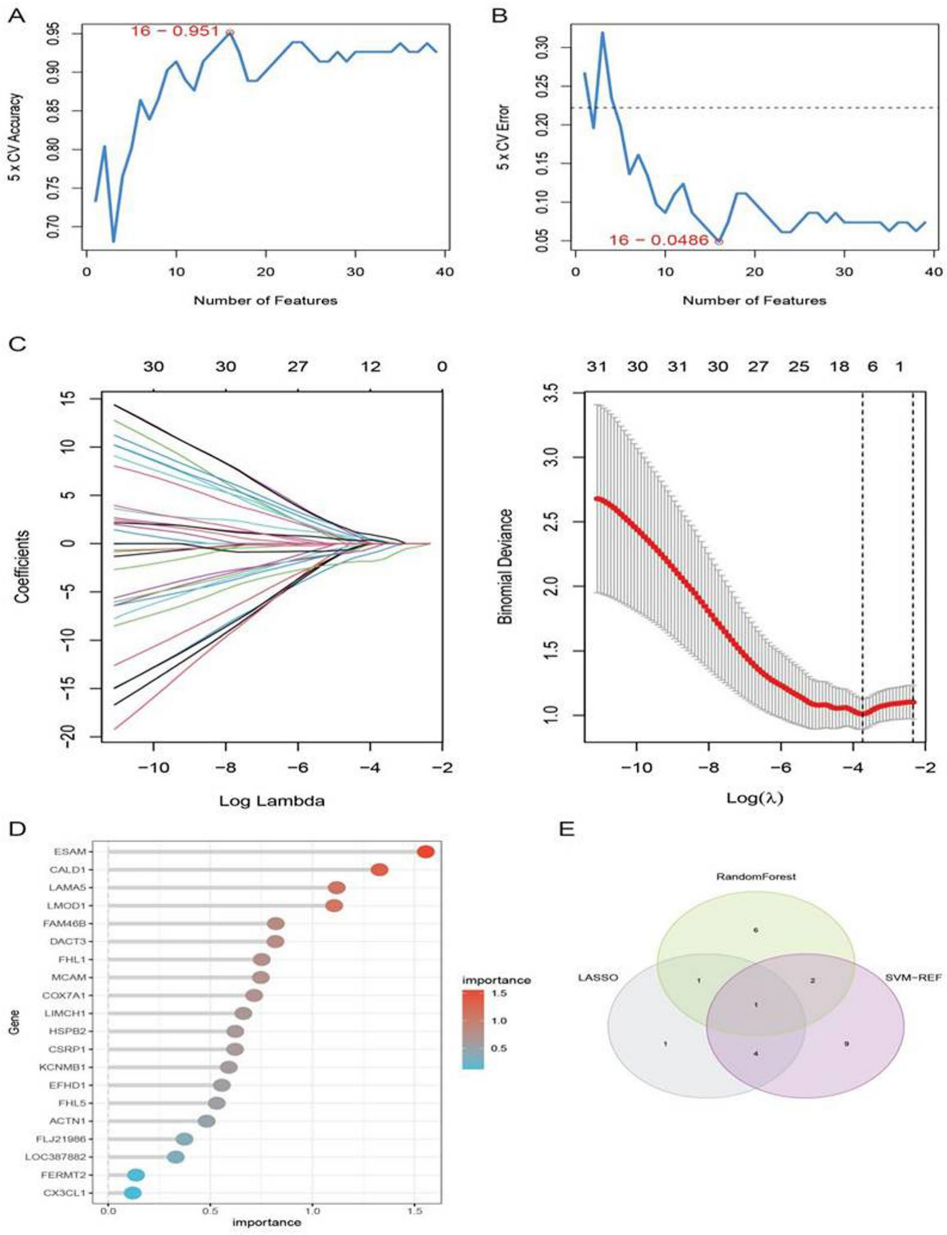
Machine Learning-Based Selection of Hub Genes Related to Neutrophils in AAA. **A** Relationship between the number of gene features and model accuracy in the SVM-REF algorithm. **B** Relationship between the number of gene features and model error rate in the SVM-REF algorithm. **C** LASSO regression analysis displaying regression coefficients of genes under different Lambda values. **D** Importance ranking of genes in the RF model, with a visualization of the top 20 genes. **E** Three machine learning algorithms selected the intersection of feature genes (SVM-REF, LASSO, RF)

To further identify key hub genes associated with neutrophil infiltration, we utilized three machine learning algorithms, SVM-REF, LASSO logistic regression, and RF model, to screen 39 differentially expressed genes. When using the SVM-REF algorithm for gene selection, the accuracy range of the model was found to be 0–0.951, with the highest accuracy point marked by a red circle (Fig. 4A), and the error rate range was 0.0486–0.35, with the lowest error rate point marked by a red circle (Fig. 4B). Therefore, the top 16 genes in SVM-REF were selected as feature genes. LASSO regression analysis revealed that when reaching the optimal λ value, 7 feature genes were selected (Fig. 4C). The RF model ranked all genes according to their importance, with the top 20 most important genes identified. The top 10 genes were selected as feature genes for the RF model (Fig. 4D). Intersection analysis of the feature genes obtained from SVM-REF, LASSO logistic regression, and RF model revealed that all three algorithms identified the NEXN gene as a feature gene (Fig. 4E). In summary, by applying multiple machine-learning algorithms to screen for key hub genes associated with neutrophil infiltration, the NEXN gene was consistently identified as a critical feature gene. It provides important clues for further research on the role of NEXN in the pathogenesis of AAA.

### Analysis of the expression and potential functions of NEXN in AAA

**Fig. 5:**
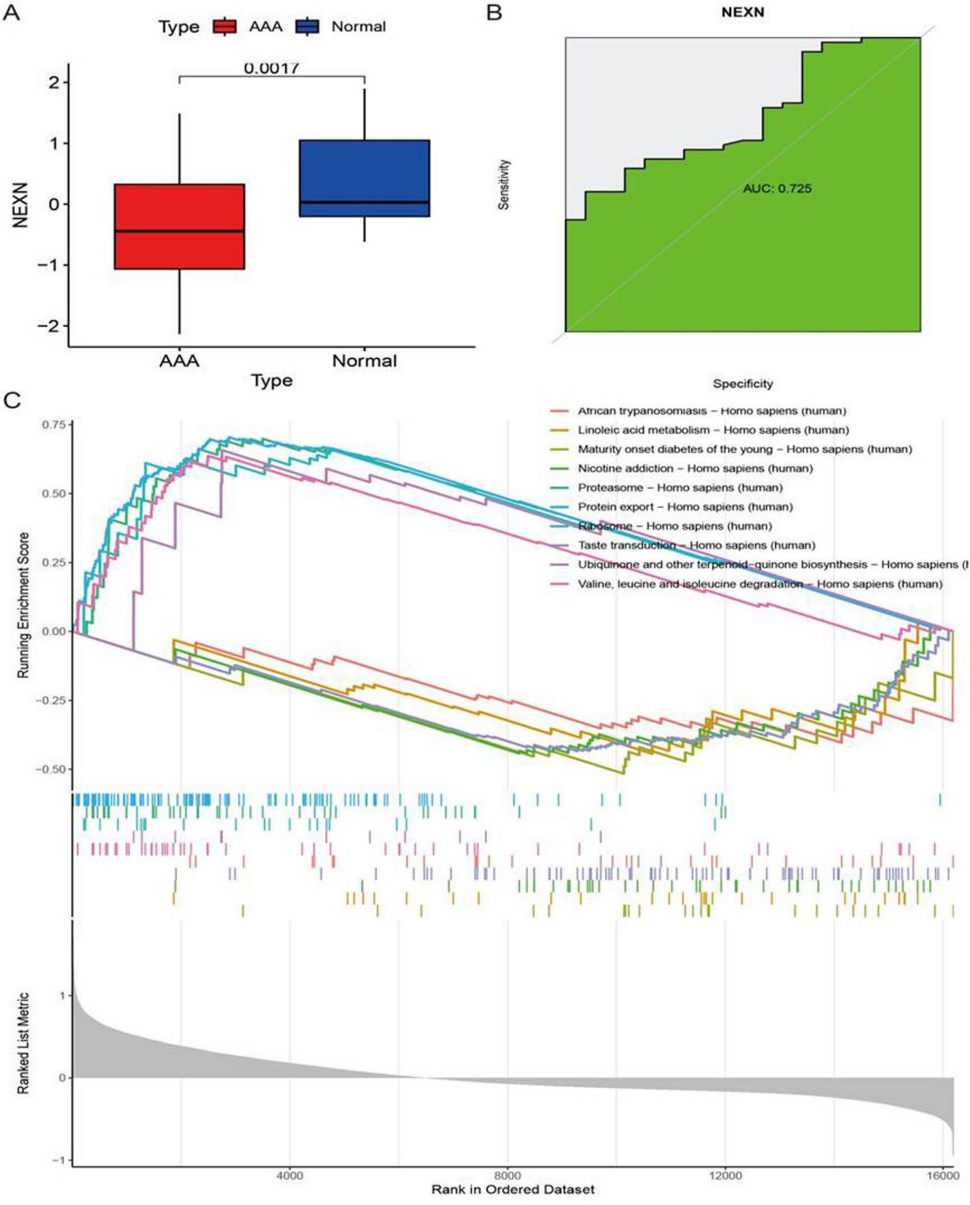
Expression, Diagnostic, and Functional Analysis of NEXN in AAA. **A** Comparison of NEXN expression levels in AAA and normal tissues. **B** ROC curve for NEXN distinguishing between AAA and normal tissues, with an AUC value of 0.725. **C** GSEA enrichment analysis displaying pathways associated with upregulation and downregulation of NEXN

In order to further investigate the expression and function of the NEXN gene in AAA, we conducted expression level analysis, ROC curve analysis, and GSEA. Compared to the control group, NEXN expression was significantly downregulated in AAA tissues (Fig. 5A). ROC curve analysis revealed that the AUC for NEXN in distinguishing AAA tissues from normal tissues was 0.725, indicating its potential for diagnosing AAA (Fig. 5B). The results of GSEA enrichment analysis indicated that in AAA, upregulation of NEXN was associated with activation of pathways such as Proteasome, Protein export, Ribosome, Ubiquinone and other terpenoid-quinone biosynthesis, and Valine, leucine, and isoleucine degradation, while downregulation of NEXN was associated with activation of pathways such as African trypanosomiasis, Linoleic acid metabolism, Maturity onset diabetes of the young, Nicotine addiction, and Taste transduction (Fig. 5C). In summary, NEXN exhibits downregulated expression in AAA tissues and has potential utility in diagnosing AAA. GSEA analysis further revealed multiple potential biological functions of NEXN in AAA, offering a new perspective for understanding the pathogenesis of AAA.

### Correlation analysis of the relationship between NEXN and immune cell infiltration in AAA

**Fig. 6:**
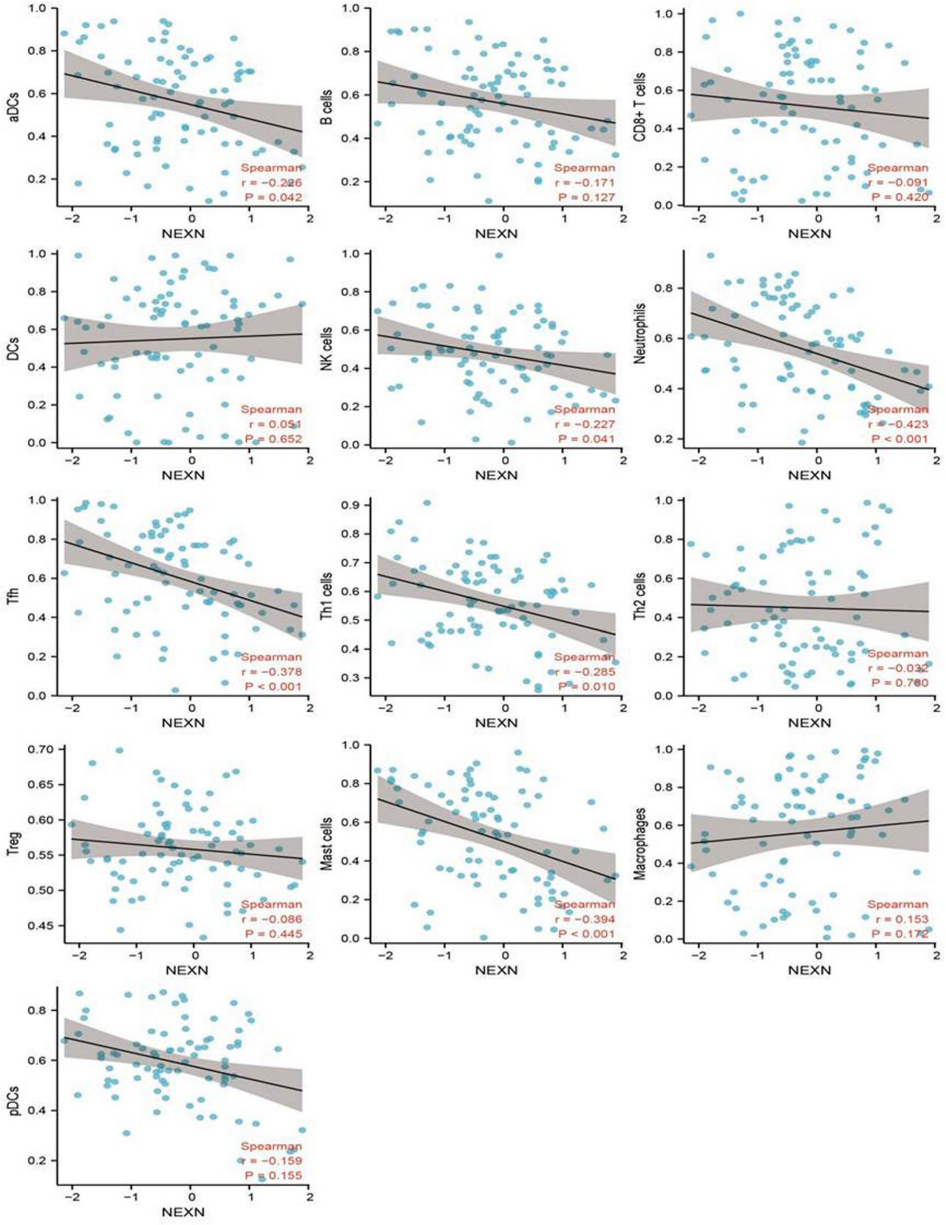
Analysis of the Negative Correlation Between NEXN and Immune Cell Infiltration in AAA. Spearman correlation analysis calculates the correlation of NEXN with various immune cells in AAA tissues

To further understand the role of the NEXN gene in AAA, we investigated its correlation with immune cell infiltration to reveal its potential impact on the immune microenvironment of AAA. The results showed that in AAA, the expression of NEXN was significantly negatively correlated with the infiltration of various immune cell types. Specifically, the expression of NEXN was negatively correlated with dendritic cells (aDCs) (R = − 0.226;* p* < 0.05), NK cells (R = − 0.227;*p* < 0.05), neutrophils (R = − 0.423; *p* < 0.05), T follicular helper cells (TFH) (R = − 0.378; *p* < 0.05), T helper type 1 cells (TH1 cells) (R = − 0.285; *p* < 0.05), and mast cells (R = − 0.394; *p* < 0.05) (Fig. 6). In conclusion, the expression of NEXN in AAA is negatively correlated with the infiltration of various key immune cells, suggesting that it may play an important role in the immune regulation process of AAA. This finding provides a new clue for further research on the immunological function of NEXN in AAA.

### The expression of NEXN in AAA tissues and its association with immune cell

**Fig. 7:**
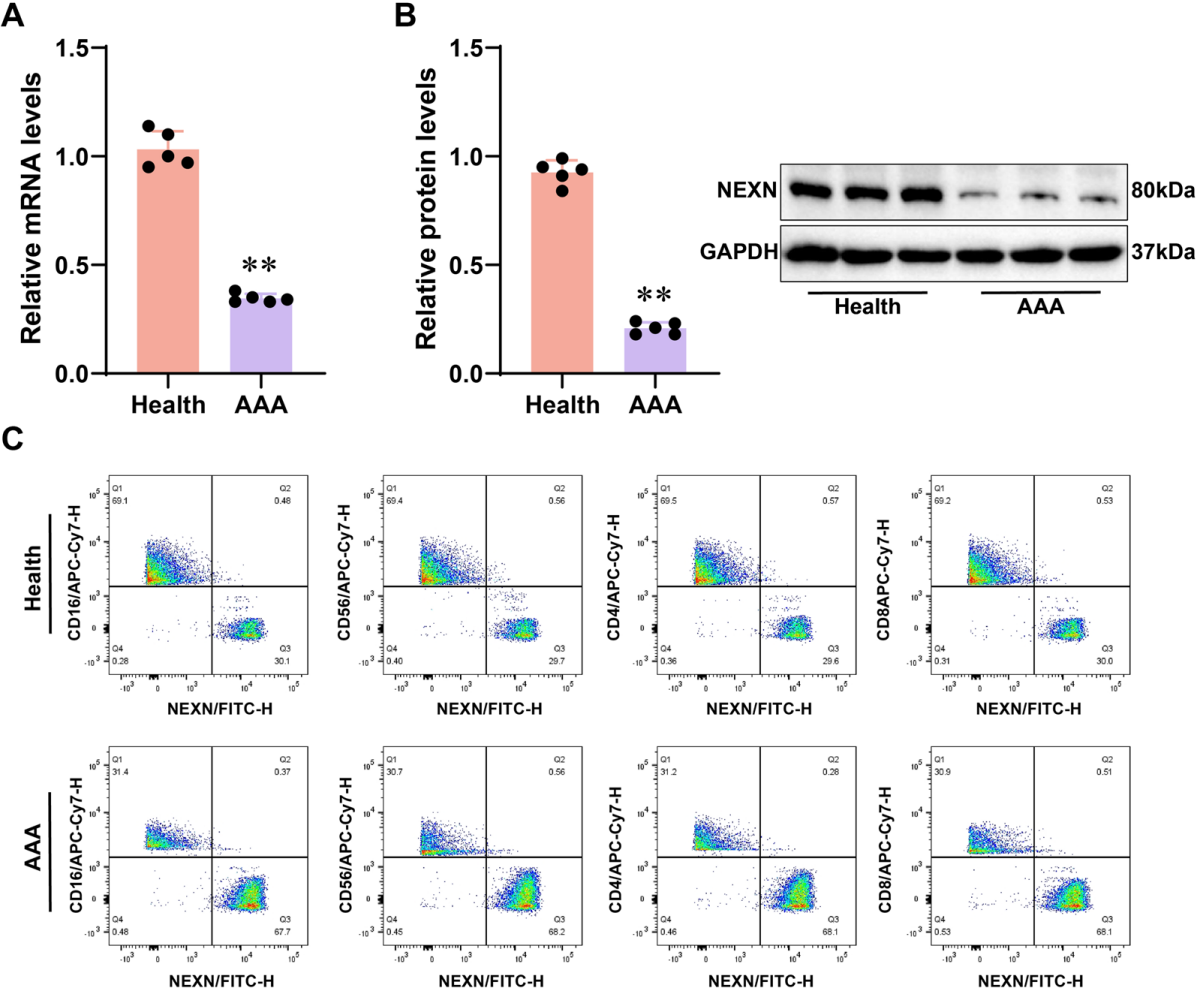
Expression of NEXN in AAA Tissues and Its Association with Immune Cell Infiltration. **A** qRT-PCR detection of mRNA expression levels of NEXN in AAA tissues (n = 5) and healthy control tissues (n = 5). **B** WB analysis of protein expression levels of NEXN in AAA and healthy control tissues. **C** FACS analysis of the relationship between NEXN expression levels and immune cell infiltration. *p < 0.05

To investigate the expression levels of NEXN in AAA and its relationship with immune cell infiltration, we utilized qRT-PCR and WB analyses to examine the mRNA and protein expression of NEXN and further studied its correlation with immune cell infiltration using FACS. Through qRT-PCR and WB analyses, we found that the mRNA and protein expression levels of NEXN in AAA tissues were significantly lower than those in the healthy control group (*p* < 0.05) (Fig. 7A–B), suggesting that NEXN may play a negative regulatory role in the occurrence and development of AAA. Further analysis using FACS demonstrated that the expression levels of NEXN in AAA tissues were significantly negatively correlated with the infiltration of immune cells such as neutrophils and NK cells (*p* < 0.05) (Fig. 7C), indicating that NEXN may be involved in the pathological process of AAA by impacting immune cell infiltration. In conclusion, NEXN is significantly downregulated in AAA tissues and is negatively correlated with the infiltration of various immune cells, suggesting that it may play a crucial role in the occurrence and development of AAA by regulating immune cell infiltration. This discovery provides a new perspective for further researching the function and potential mechanisms of NEXN in AAA.

### The impact of NEXN on cell proliferation, migration, and invasion capabilities

**Fig. 8:**
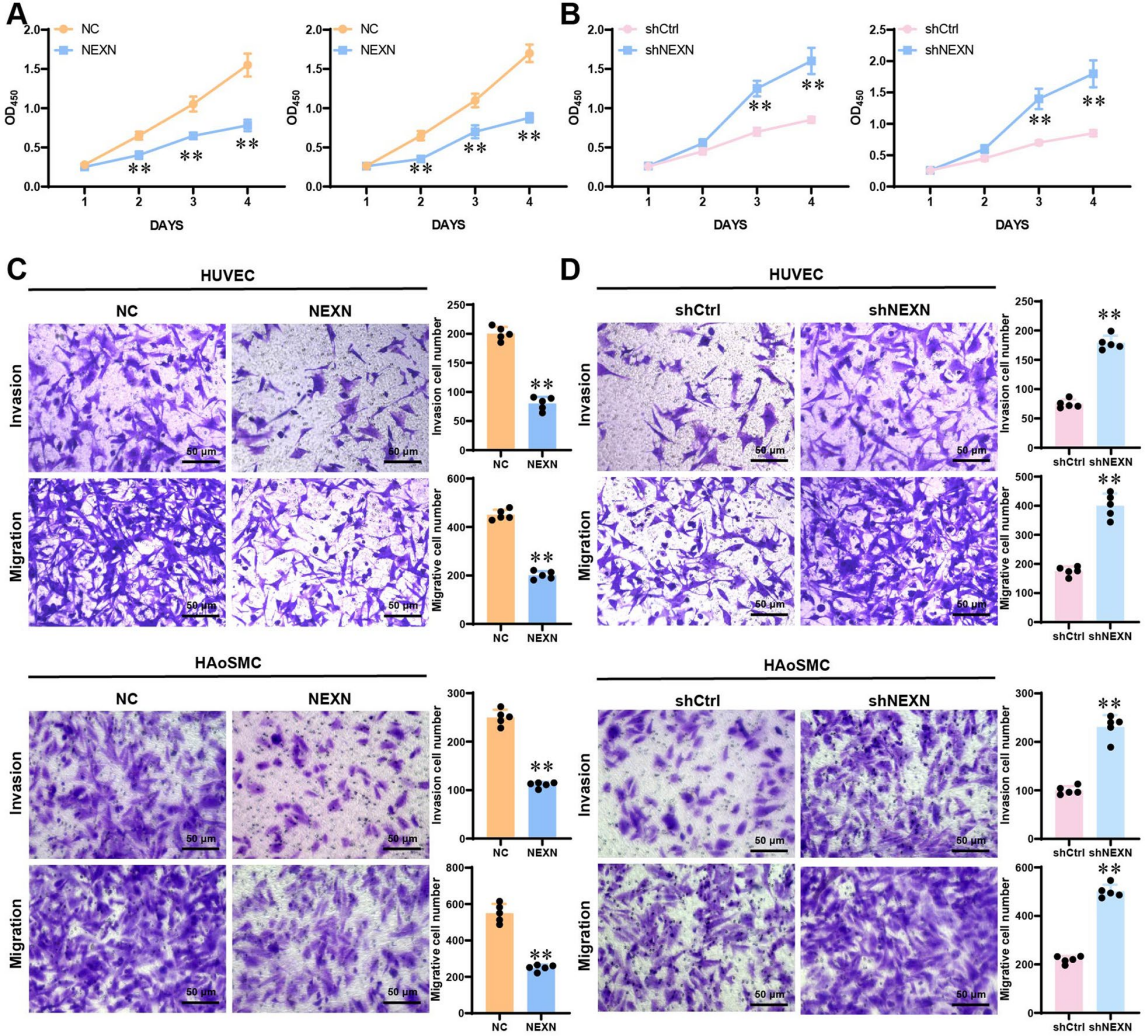
Impact of NEXN on Cell Functions. **A** CCK-8 assay detecting the effect of NEXN overexpression on the proliferation ability of HUVECs and HAoSMCs. **B** CCK-8 assay assessing the impact of NEXN knockout on the proliferation ability of HUVECs and HAoSMCs. **C** Transwell migration and invasion experiments evaluate NEXN overexpression's influence on cell migration and invasion capabilities. **D** Transwell migration and invasion assays investigating the effect of NEXN knockdown on cell migration and invasion abilities. *p < 0.05, **p < 0.01, ***p < 0.001. Cell experiments were performed in triplicate

To study the effect of NEXN on cell function, we overexpressed and knocked out NEXN in HUVEC and HAoSMC cells using lentiviral vectors and CRISPR-Cas9 gene editing technology, respectively, and evaluated their impact on cell proliferation, migration, and invasion capabilities. CCK-8 assay results showed that overexpression of NEXN significantly inhibited the proliferation ability of HUVEC and HAoSMC cells (*p* < 0.05) (Fig. 8A), while knockout of NEXN led to a significant increase in cell proliferation capability (*p* < 0.05) (Fig. 8B), indicating that NEXN plays a role in inhibiting cell proliferation related to AAA. Results from Transwell migration and Matrigel invasion assays demonstrated that overexpression of NEXN significantly inhibited the migration and invasion capabilities of HUVEC and HAoSMC cells (*p* < 0.05) (Fig. 8C); conversely, knockout of NEXN led to a significant increase in cell migration and invasion capabilities (*p* < 0.05) (Fig. 8D), indicating that NEXN plays a crucial inhibitory role in cell migration and invasion processes. In conclusion, NEXN exerts a key role in regulating cell proliferation, migration, and invasion capabilities in AAA-related cell functions. These findings provide a theoretical basis for further research on the biological functions of NEXN in AAA and its potential therapeutic value.

### The inhibitory effect of NEXN in the AAA model of ApoE^−/−^ mice

**Fig. 9:**
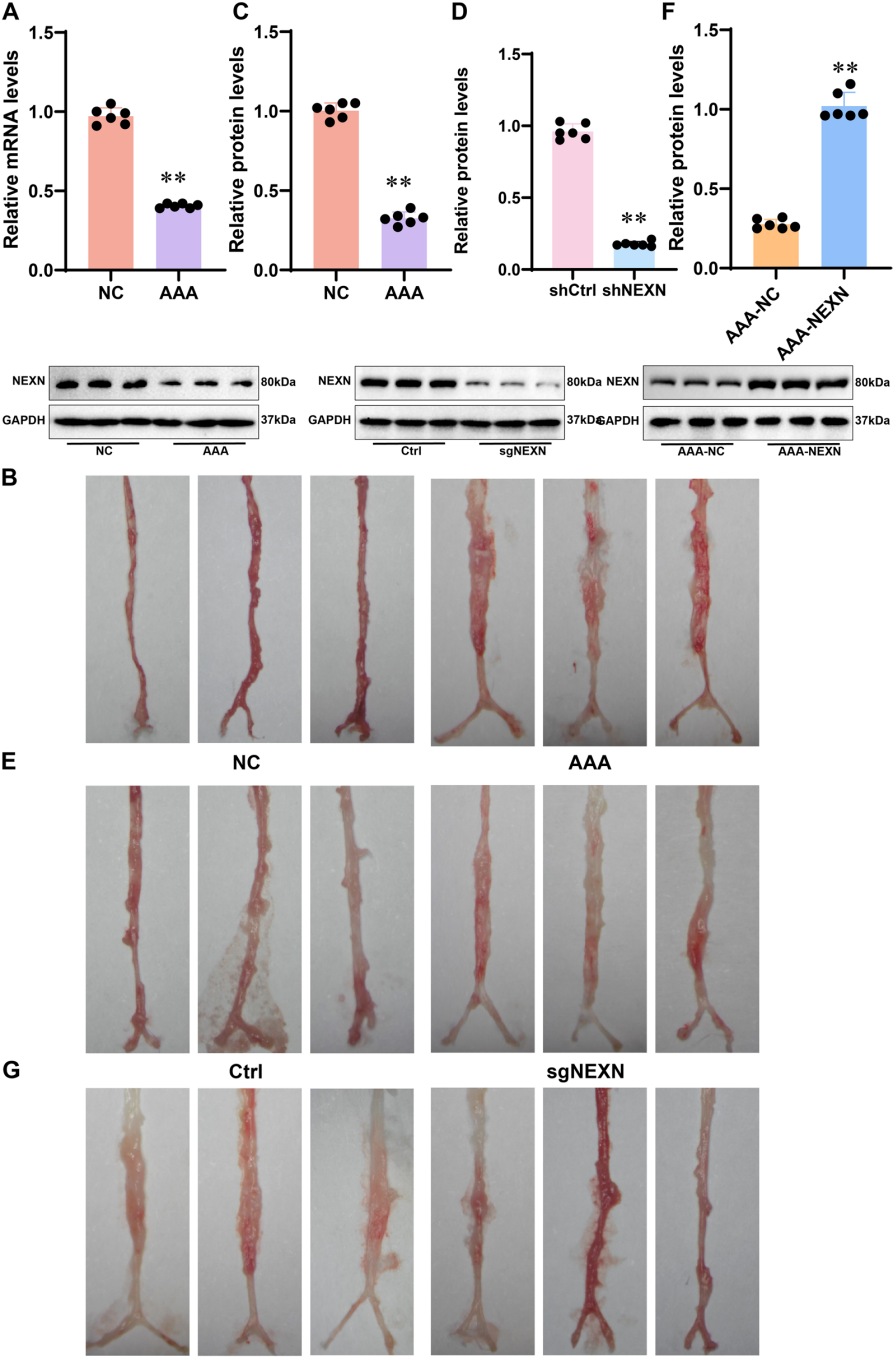
Role of NEXN in the AAA Mouse Model. **A** qRT-PCR assessment of NEXN mRNA expression levels in the AAA mouse model. **B** Morphology of the AAA model. **C** WB analysis of NEXN protein expression levels in the AAA mouse model. **D** WB analysis of NEXN protein expression levels in the NEXN knockout normal group. **E** Morphology of the NEXN knockout normal group. **F** WB analysis of NEXN protein expression levels in the AAA group with NEXN overexpression. **G** Morphology of the AAA model with NEXN overexpression. *p < 0.05. N = 6

To further verify the role of NEXN in AAA, we utilized the ApoE^−/−^ mouse model to investigate changes in NEXN expression and its impact on the severity of AAA through in vivo experiments. The proportion of aneurysm formation is 80%. Through qRT-PCR and WB analyses, a significant decrease in the expression levels of NEXN was observed in the ApoE^−/−^ mouse AAA model group (p < 0.05), indicating the successful establishment of the AAA model (Fig. 9A–C), further validating the results of in vitro experiments. Knocking out NEXN in normal mice significantly worsened the severity of AAA (*p* < 0.05) and increased the diameter of the aorta (Fig. 9D−E). Conversely, overexpressing NEXN in ApoE^−/−^ mice significantly reduced the severity of AAA (*p* < 0.05) and decreased the aortic diameter (Fig. 9F–G), demonstrating the inhibitory role of NEXN in the progression of AAA. In conclusion, NEXN exhibits a significant inhibitory effect in the ApoE^−/−^ mouse AAA model. This finding provides a new potential target for the treatment of AAA and serves as an important basis for further research on the function and mechanism of NEXN in AAA.

### Mechanistic study of NEXN in the AAA model of ApoE^−/−^ Mice

**Fig. 10:**
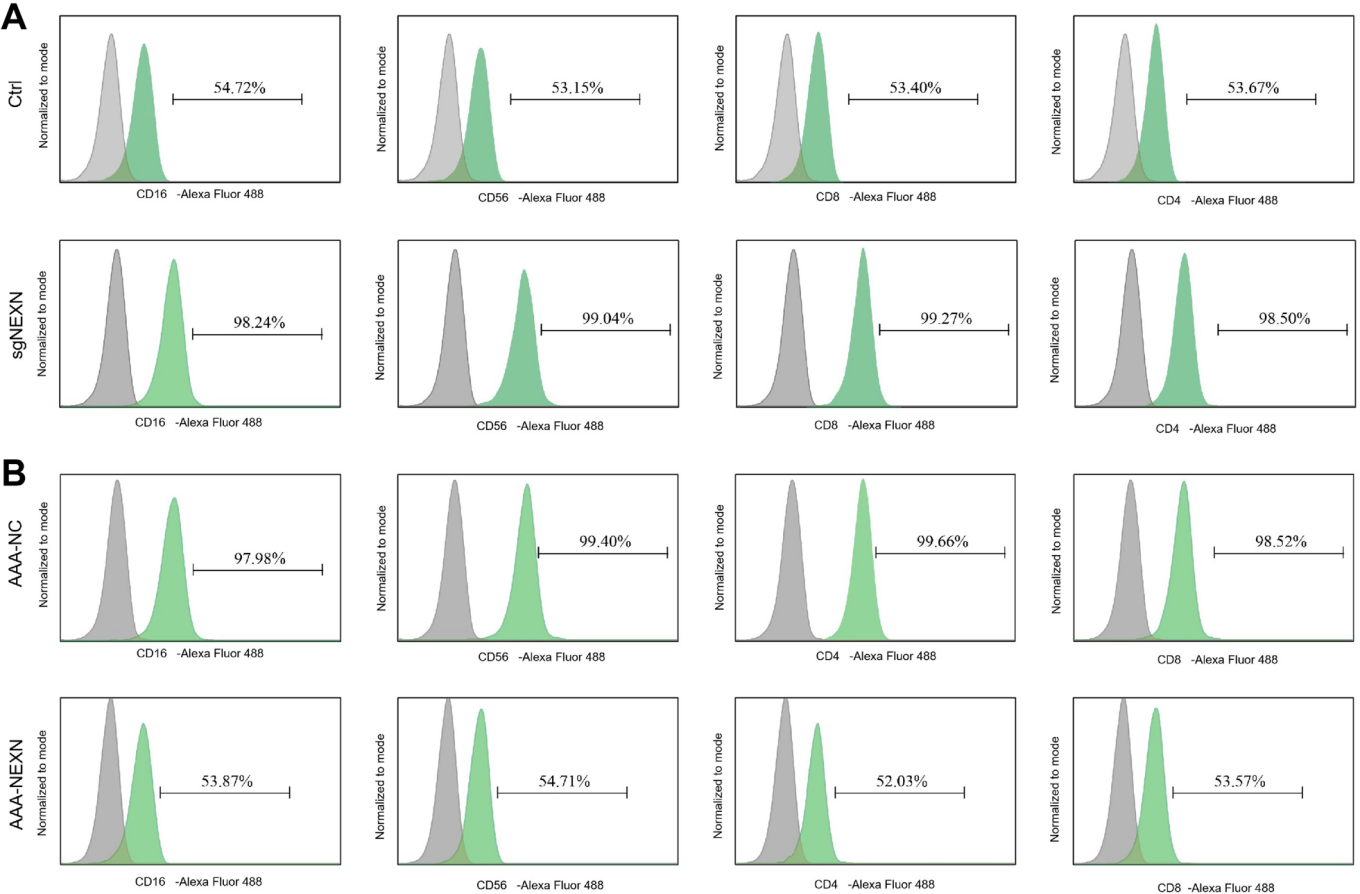
Relationship Between NEXN Expression Levels and Immune Cell Infiltration Analyzed by FACS. **A** Flow cytometry analysis of the proportions of neutrophils (CD16), NK cells (CD56), effector T cells (CD8), and effector B cells (CD4) in the control group (Ctrl) and NEXN knockdown group (sgNEXN). **B** Flow cytometry analysis of the proportions of CD16, CD56, CD8, and CD4 cells in the NEXN control group (AAA-NC) and NEXN overexpression group (AAA-NEXN). Each group with n = 6. Data presented as mean ± standard error analyzed using a t-test, with * indicating *p* < 0.05

To further validate the role of NEXN in AAA, we used the ApoE^−/−^ mouse model to explore changes in NEXN expression and its impact on the severity of AAA lesions through in vivo experiments. Flow cytometry analysis revealed a significant increase in the infiltration of neutrophils, NK cells, effector T cells, and effector B cells in NEXN knockout mouse models (*p* < 0.05) (Fig. 10A); while in NEXN overexpression mouse models, the infiltration of these immune cells significantly decreased (Fig. 10B), suggesting that NEXN may influence the development of AAA by regulating immune cell infiltration. In conclusion, the significant inhibitory mechanism of NEXN in the ApoE^−/−^ mouse AAA model may be related to regulating immune cell infiltration.

## Discussion

If not diagnosed or treated promptly, the natural course of AAA involves growth and eventual rupture (Schmitz-Rixen et al. 2020, Menges et al. 2023). The mortality rate from AAA rupture is extremely high, making it one of the leading causes of death worldwide (Anagnostakos and Lal 2021; Schmitz-Rixen et al. 2020, Gao et al. 2023). Identifying new biomarkers and potential mechanisms is crucial for treating AAA patients. In this study, considering the significance of immune cell infiltration in AAA pathology, we combined ssGSEA, WGCNA, and differential expression analysis to identify neutrophil-related genes involved in AAA. Using machine learning algorithms, we identified NEXN as a core gene with diagnostic potential for AAA. Our findings show a significant increase in the infiltration of neutrophils, NK cells, and pDCs in AAA tissues, consistent with previous literature on the role of immune cells in AAA. Through bioinformatics and experimental validation, we further elucidated the critical regulatory role of NEXN in this process.

Neutrophils, as major inflammatory cells, play a crucial role in the pathogenesis of AAA (Yuan et al. 2021, Xu et al. 2023; Hadzikadunic et al. 2023). Previous studies have shown that neutrophils promote the development of AAA by releasing enzymes and reactive oxygen species, leading to tissue damage and inflammatory responses (Xiao et al. 2021; Voynow and Shinbashi 2021; Cui et al. 2021). In this study, WGCNA analysis identified 234 genes related to neutrophil infiltration, enriching our understanding of the mechanisms by which neutrophils contribute to AAA. Unlike other studies, we revealed the increased infiltration of neutrophils and systematically analyzed the specific functions and mechanisms of these genes in AAA.

In this study, we identified 39 significantly differentially expressed genes through differential expression analysis and conducted GO and KEGG functional enrichment analyses. The results showed that these genes play important roles in actin-related BP and signaling pathways. Unlike previous studies that provided only simple descriptions of AAA-related genes, our functional enrichment analysis and PPI network construction systematically revealed the functions and mechanisms of these genes. It not only deepens our understanding of the molecular mechanisms underlying AAA but also provides new directions for future research.

Using various machine learning algorithms, we ultimately identified NEXN as a key hub gene related to neutrophil infiltration. The identification of NEXN and its unique role in AAA is a significant highlight of this study. Unlike other AAA-related genes discovered in previous research, NEXN is notably downregulated in AAA tissues and negatively correlates with immune cell infiltration. Further experimental validation confirmed this role of NEXN, indicating that its downregulated expression in AAA tissues may influence the progression.

To further validate the function of NEXN in AAA, we conducted in vivo experiments using an ApoE^−/−^ mouse model. The results showed that NEXN inhibits the progression of AAA in these mice by regulating immune cell infiltration. This finding contrasts sharply with previous studies on the role of NEXN in other diseases, highlighting its unique regulatory mechanism in AAA. Our research not only provides new experimental evidence but also offers fresh perspectives and potential applications for NEXN in AAA.

This study’s innovation lies in the combination of WGCNA and machine learning methods to systematically investigate neutrophil infiltration in AAA tissues and identify the hub gene NEXN. We not only uncovered key molecular mechanisms in the pathogenesis of AAA but also proposed new potential therapeutic targets. As a novel potential biomarker, NEXN holds significant promise for the diagnosis and treatment of AAA. Our findings provide a new theoretical foundation and clinical guidance for the early diagnosis and personalized treatment of AAA.

This study has several limitations. First, Although Ang II-induced ApoE-/- mice mainly develop dissecting aneurysms, this model provides key insights into acute inflammation and vascular remodeling. The observed gene expression changes reflect AAA's acute phase, significantly activating inflammation, vascular remodeling, and smooth muscle apoptosis pathways. Despite not fully replicating chronic true aneurysms, it remains a widely used model for early molecular events in aneurysm progression. Second, the dataset lacks clinical information, preventing the consideration of key risk factors such as sex and smoking status. Third, the interaction between NEXN and immune cells, as well as its regulatory role in AAA, requires further in vitro and in vivo validation. Lastly, the potential of NEXN as a biomarker and therapeutic target needs verification in clinical settings. Future research will focus on: (1) analyzing NEXN expression at different AAA stages via WB/qPCR; (2) examining the correlation between NEXN expression and neutrophil infiltration in AAA models, along with immunofluorescence-based co-localization analysis; (3) using CRISPR-based knockdown/overexpression models to investigate its role in AAA and inflammation; and (4) performing metabolic analyses to assess its impact on amino acid metabolism. This study employed only the PPE-induced AAA model without comparisons to alternative models such as calcium chloride-induced AAA. Future research should determine whether NEXN remains a key gene across AAA models.

Future research should further explore the role and mechanisms of NEXN, particularly in different pathological states. Multi-center, large-sample clinical studies are recommended to assess NEXN differences between AAA patients and healthy individuals to evaluate its potential for early diagnosis and treatment of AAA. Additionally, developing NEXN-targeted therapies is a promising area for further investigation. Additionally, multidisciplinary collaboration is crucial in AAA research, and integrating expertise from molecular biology, immunology, and clinical medicine is essential for advancing our understanding of AAA mechanisms and developing treatment strategies. In summary, this study uses WGCNA and machine learning methods to uncover the molecular mechanisms of neutrophil infiltration in AAA tissues and identify the hub gene NEXN, providing new potential targets for AAA diagnosis and treatment with significant scientific and clinical value.

## Conclusion

**Fig. 11:**
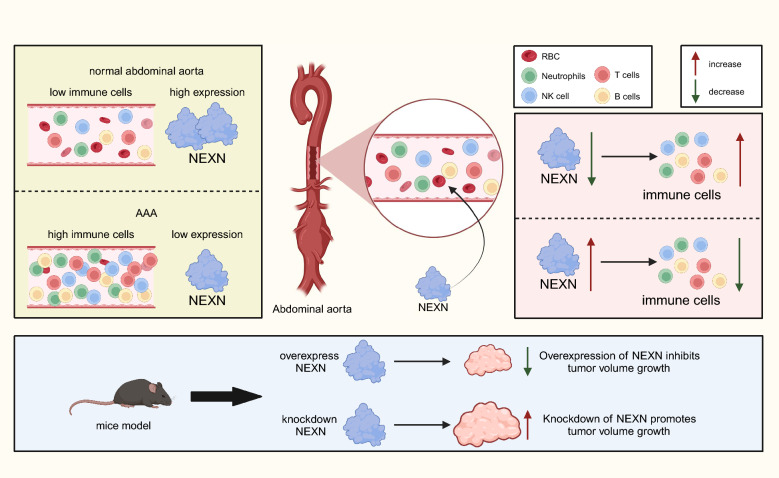
Revealing the Key Role of NEXN in AAA and Its Association with Immune Cell Infiltration

This study comprehensively analyzes immune cell infiltration in AAA tissues, revealing a significant neutrophil increase. Through WGCNA and differential gene expression analysis, hub gene modules related to neutrophil infiltration are identified. Further functional enrichment analysis and PPI network construction reveal the important roles of these genes in actin-related BP and signaling pathways. Machine learning algorithms further identify NEXN as a key hub gene in AAA, showing its downregulated expression in AAA tissues and a significant negative correlation with immune cell infiltration (Fig. 11).

The discovery of NEXN provides a new candidate target for biomarker research in AAA and offers insights for studying other inflammation-driven vascular diseases. By revealing the association between NEXN and neutrophil infiltration, this study provides a new perspective on the pathological mechanisms of AAA, enhancing our understanding of the role of immune cells in AAA progression. Functional studies of NEXN indicate that it may be inhibitory in regulating immune cell infiltration, offering a scientific basis for developing NEXN-targeted therapeutic strategies. Changes in NEXN expression can serve as a biomarker for diagnosing AAA, potentially improving early diagnostic capabilities for the disease.

## Data Availability

All data can be provided as needed.

## Abbreviations

AAA: Abdominal aortic aneurysm
ANOVA: Analysis of variance
AUC: Area under the curve
BP: Biological processes
CC: Cellular components
FACS: Fluorescence-activated cell sorting
GEO: Gene expression omnibus
GO: Gene ontology
GSEA: Gene set enrichment analysis
IHC: Immunohistochemistry
KEGG: Kyoto Encyclopedia of Genes and Genomes
MF: Molecular functions
NEXN: Nexilin
NK cells: Natural killer cells
PPI: Protein–protein interaction
RF: Random forest
ROC: Receiver operating characteristic
ssGSEA: Single-sample gene set enrichment analysis
SVM-REF: Support vector machine: recursive feature elimination
TFH: T follicular helper cells
TH1 cells: T helper type 1 cells
WB: Western blot
WGCNA: Weighted gene co-expression network analysis
XGBoost: Extreme gradient boosting
